# Morbidity, Recurrence and Survival Following Pelvic Exenteration for Gynaecological Malignancies: A Retrospective, Single-Centre Study

**DOI:** 10.3390/jcm15103957

**Published:** 2026-05-20

**Authors:** Shruti Zalawadia, Sofia Lekka, Zahra Al-Jumaili, Elly Brockbank, Ranjit Manchanda, Arjun Jeyarajah, Saurabh Phadnis, Michail Sideris

**Affiliations:** 1 Department of Gynaecologic Oncology, Royal London Hospital, Barts Health Trust, London E1 1BB, UK; s.zalawadia@nhs.net (S.Z.); s.lekka@nhs.net (S.L.); zahra.al-jumaili@nhs.net (Z.A.-J.); elly.brockbank@nhs.net (E.B.); r.manchanda@qmul.ac.uk (R.M.); arjun.jeyarajah@nhs.net (A.J.); s.phadnis@nhs.net (S.P.); 2 Wolfson Institute of Population Health, Queen Mary University of London, London EC1M 6BQ, UK; 3 Department of Health Services Research and Policy, London School of Hygiene & Tropical Medicine, London WC1H 9SH, UK; 4 MRC Clinical Trials Unit at UCL, Institute of Clinical Trials & Methodology, Faculty of Population Health Sciences, University College London, London WC1V 6LJ, UK

**Keywords:** pelvic exenteration, gynaecological malignancies, morbidity, recurrence patterns

## Abstract

**Background/Objectives**: We evaluated perioperative morbidity, recurrence patterns and survival outcomes following pelvic exenteration (PE) at a tertiary referral centre. **Methods**: A retrospective observational study was conducted in women undergoing PE from 2004 to 2024. We collected demographics, performance status (PS), comorbidities, body mass index (BMI), tumour histology, intraoperative details, postoperative morbidity (Clavien–Dindo classification), mortality, length of stay (LOS), recurrence patterns and cancer-related death. Descriptive statistics were performed alongside Kaplan–Meier survival analysis. **Results**: Forty-seven patients underwent PE; median PS was 0 [interquartile range (IQR) 0–0]. Median ages at diagnosis and surgery were 55 (IQR 49–66) and 60 (IQR 50–68) years, respectively, with a median follow-up of 26 months (IQR 12–64). Thirty-two procedures (68%) were performed for recurrent and N = 15 (32%) for primary disease. Histology included N = 17 endometrial (36%), N = 10 vulval (23%), ovarian (15%), N = 5 cervical (11%) and N = 7 vaginal (15%) cases. Eighteen patients (38%) underwent total PE, N = 15 (32%) anterior PE and N = 14 (30%) posterior PE. Median blood loss was 1.5 L (IQR 0.85–2.0) and median operative time was 391 mis (IQR 313–482). Median HDU stay was 4 days (IQR 2–5) and LOS was 17 days (IQR 13–31). One postoperative death occurred. Major complications (Clavien–Dindo ≥3) occurred in 15 patients (32%). Late complications occurred in n = 17 (36.2%) women. Nineteen patients (41%) remained recurrence-free; N = 4 (9%) developed local and N = 24 (51%) distant recurrence. Mean overall survival time post-surgery for curative intent PE (N = 46) was 94 months (95%CI = 57–131 months); for primary tumours this was 51.6 (95%CI = 31–72) vs. 99 (56.01–142) for recurrent disease (*p* > 0.05). **Conclusions**: Pelvic exenteration is associated with acceptable morbidity and mortality in carefully selected patients, offering excellent locoregional disease control.

## 1. Introduction

Originally described by Brunschwig in 1948 as a palliative operation for symptom relief in advanced cervical cancer [1], pelvic exenteration (PE) has since evolved into a potentially curative procedure in carefully selected patients. PE refers to the radical en bloc multi-visceral resection of pelvic structures, indicated for locally advanced, persistent, or recurrent disease, involving cervical, endometrial, vaginal, vulvar, and ovarian carcinomas [2]. Despite advances in surgical techniques, perioperative management, and reconstructive approaches, PE remains one of the most complex and radical procedures in gynaecological oncology (GO) often regarded as the final surgical option when other treatments are exhausted or not feasible [3]. Improvements in surgical planning, imaging, and multidisciplinary perioperative care have contributed to better operative outcomes and reduced perioperative mortality.

Nevertheless, pelvic exenteration continues to be associated with substantial morbidity. Reported postoperative complication rates range between 30% and 60% [4,5]. Perioperative mortality is approximately 5%, with sepsis representing the leading cause [6,7]. These findings signify the importance of careful patient selection.

Despite its associated morbidity, pelvic exenteration can provide meaningful oncological benefit for selected patients [8]. The earlier literature reports 5-year OS rates following curative intent PE to be 30.8%, ranging between 30% and 55% [7,8,9]. Prognosis is strongly influenced by several factors, including lymph node status, pelvic sidewall involvement, and the ability to achieve complete tumour resection with negative margins [10]. Additional adverse prognostic factors associated with poorer disease-free survival include extent of PE (total PE), presence of positive surgical margins or lymphovascular space invasion (LVSI) [8].

Given the surgical complexity and the importance of patient selection, outcomes following PE are highly dependent on surgical expertise, multidisciplinary collaboration, and institutional experience. For this reason, tertiary referral centres with specialised GO services are set up to safely provide this surgical option and should evaluate their long-term outcomes. Therefore, reporting and disseminating institutional experiences remains essential to further understand perioperative morbidity, recurrence patterns, and survival outcomes associated with this procedure.

The aim of this study was to describe our local institutional experience with PE. We report perioperative morbidity, recurrence patterns, and survival outcomes in women who underwent PE for advanced or recurrent gynaecological malignancies with curative or palliative intent.

## 2. Materials and Methods

### 2.1. Study Design and Patient Selection

A retrospective cohort study was conducted including all identified women who underwent pelvic exenteration (PE) for primary or recurrent gynaecological malignancies at a tertiary referral centre in London. All cases were identified from our institutional surgical (PE) databases from August 2004 to October 2024. Eligible women included those undergoing anterior, posterior, or total pelvic exenteration with curative or palliative intent for any advanced primary or recurrent histologically confirmed gynaecological malignancies.

### 2.2. Inclusion Criteria

Women undergoing anterior, posterior, or total PE with curative or palliative intent for histologically confirmed primary or recurrent gynaecological malignancies at our tertiary centre from August 2004 to October 2024.

### 2.3. Exclusion Criteria

We excluded patients undergoing palliative debulking procedures not classified as PE; cases with incomplete operative records; and cases identified outside the formal PE database who could not be verified. We also excluded cases performed in 2025 to achieve at least 12 months of follow-up.

### 2.4. Data Collection

Clinical data were extracted from electronic patient records (Millenium Cerner). We used a prospectively designed MS Excel spreadsheet. Variables of interest included patient demographics (age, body mass index, comorbidities), Eastern Cooperative Oncology Group Performance Status (ECOG-PS), tumour histology, disease status at surgery (primary versus recurrent disease), prior oncological treatment including chemotherapy and radiotherapy.

We also collected data on intraoperative variables including type of pelvic exenteration (anterior, posterior, or total), operative time, estimated blood loss, and reconstruction approach (primary closure versus flap reconstruction). We included postoperative outcomes such as need and length of stay on high dependency unit (HDU), length of hospital stay, and postoperative morbidity (30-day) using Clavien–Dindo (CD) scale and mortality (CD5); we also included late complications (>30 days).

Postoperative complications were graded using the Clavien–Dindo classification system. Major complications were defined as grade III or higher. Oncological outcomes included recurrence patterns (local or distant), and overall survival (OS). The DFI was defined exclusively for patients who underwent PE for recurrent gynaecological tumours and reflects the interval from completion of the last prior treatment to the date of PE. Follow-up duration was calculated from the date of PE to the last clinical review or death.

### 2.5. Statistical Analysis

Continuous variables were summarised using medians and interquartile ranges (IQRs), and categorical variables using frequencies and percentages. Survival outcomes following surgery were estimated using the Kaplan–Meier method. Overall survival was defined as the time from the date of surgery to death from any cause. Patients who were alive at the last follow-up were censored at that time. Differences in survival between groups were assessed using the log-rank test. Statistical analyses were conducted using IBM SPSS Statistics version 29 (IBM Corp., Armonk, NY, USA). A *p*-value < 0.05 was considered statistically significant.

## 3. Results

### 3.1. Patient Cohort and Baseline Characteristics

**Figure 1:**
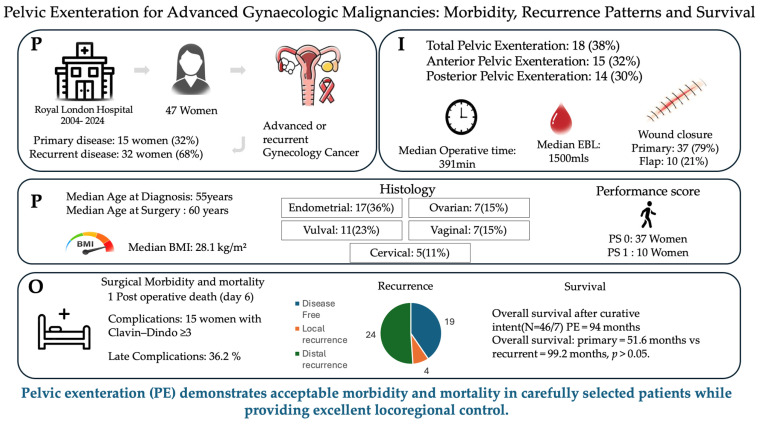
Infographic representation of cohort summary and key outcomes. P = population, I = Intervention, O = Outcomes.

Between August 2004 and December 2024, 47 patients underwent PE for gynaecological malignancies at our tertiary centre. Median age at diagnosis was 55 (IQR 49–66) and at the time of surgery 60 years (IQR 50–68). The median ECOG Performance Status (PS) was 0 (IQR 0–0), with 37 patients (78.7%) classified as PS 0 and 10 (21.3%) as PS 1. Median body mass index (BMI) was 28.1 kg/m^2^ (IQR 19.7–53.0). Figure 1’s infographics summarise the patient cohort and key results.

Comorbidities included vasculopathy in four patients (8.5%) and hypertension in 20 (42.6%). Six patients (12.8%) were active smokers and eight (17.0%) were ex-smokers.

Thirty-two procedures (68.0%) were performed for recurrent and 15 (32.0%) for primary tumours. Tumour distribution included endometrial (17/47, 36.2%), vulvar (11/47, 23.4%), ovarian (7/47, 14.9%), vaginal (7/47, 14.9%), and cervical cancers (5/47, 10.6%). Most procedures were performed with curative intent (46/47, 97.9%), with one case (2.1%) undertaken for palliation of symptoms.

Prior treatment varied across the cohort with N = 6 patients (12.8%) having undergone previous total hysterectomy ± pelvic lymph node dissection, N = 7 (14.9%) total hysterectomy with bilateral salpingo-oophorectomy and brachytherapy ± external beam radiotherapy, and N = 3 (6.4%) radical hysterectomy with adjuvant therapy. Eight patients (17.0%) had received prior radiotherapy and/or chemotherapy. Sixteen patients (34.0%) had no prior treatment before PE. Baseline patient characteristics and related outcomes are summarised in Table 1.

### 3.2. Intraoperative Details

Total PE (TPE) was performed in N = 18 patients (38%), anterior PE (APE) in N = 15 (32%), and posterior PE (PE) in N = 14 (30%). Median operative time was 391 min spanning from 313 to 482 min, and median estimated blood loss (EBL) was 1.5 L (IQR 0.85–2.0). Additionally, N = 32 of patients (68%) received intraoperative blood transfusion.

A systematic pelvic lymphadenectomy was performed in N = 5 patients (10.6%) and combined pelvic and para-aortic lymphadenectomy in N = 7 patients (14.9).

Flap reconstruction was performed in N = 10/47 of cases (21%), while the remainder underwent primary closure.

Urinary diversion was performed with urinary ileal conduit in N = 29 patients (61.7%), followed by jejunum conduit in two patients (4.2%) and formation of a Miami pouch in one patient (2.1%).

One patient underwent neovaginal reconstruction. Omental flap was performed in N = 26 out of 47 cases (55%) to minimise surgical morbidity following a radical surgical procedure.

### 3.3. Histopathology Outcomes

LVSI was present in N = 13 patients (27.7%), absent in N = 31 (66.0%), and not reported in N = 3 cases (6.4%). Surgical margins were negative (R0) in N = 39 patients (83.0%), while N = 5 (10.6%) had close margins (<9 mm). One patient (2.1%) had microscopically involved margins, and one (2.1%) macroscopic residual disease.

### 3.4. Postoperative Outcomes and Morbidity

Median high dependency unit (HDU) stay was 4 days (IQR 2–5), and median length of hospital stay was 17 days (IQR 13–31). Postoperative complications occurred in N = 41 patients (87.2%). According to the Clavien–Dindo (CD) classification, N = 25 patients (53.2%) experienced minor complications (grade I–II), while N = 15 (31.9%) developed major complications (grade ≥ III). One postoperative death occurred (1/47, 2.1%) on postoperative day 6 due to sepsis.

One patient (2.1%) required return to theatre on postoperative day 2 for repair of an ileal conduit leak.

Late complications were observed in N = 17 patients (36.2%), including enterovaginal fistula (N = 1/47, 2.1%), wound complications (N = 3/47, 6.4%), urinary complications (N = 4/47, 8.5%), persistent pelvic collections (N = 3/47, 6.4%), infections requiring readmission (N = 2/47, 4.3%), stoma necrosis requiring refashioning (N = 2/47, 4.3%), and deep vein thrombosis (N = 2/47, 4.3%).

### 3.5. Oncological Outcomes

Median DFI prior to exenteration (for recurrent tumours only) was 2 years (IQR 0–2). Median follow-up was 26 months (IQR 12–64); mean value for follow-up was 46 months with an SD of 51. The discrepancy between mean and median (26 months, IQR 12–64) follow-up reflects significant right-skew attributable to increasing case volume in recent years. Overall, N = 19 patients (41%) remained recurrence-free. Local recurrence occurred in N = 4 patients (9%), while distant recurrence occurred in N = 24 patients (51%).

Mean overall survival time for curative intent PEs was 94 months (95%CI = 57–131 months). For primary tumours only, mean survival post-PE was 51.6 (95%CI = 31–72) vs. 99.2 (56.01–142) for recurrent tumours (*p* > 0.05).

**Figure 2:**
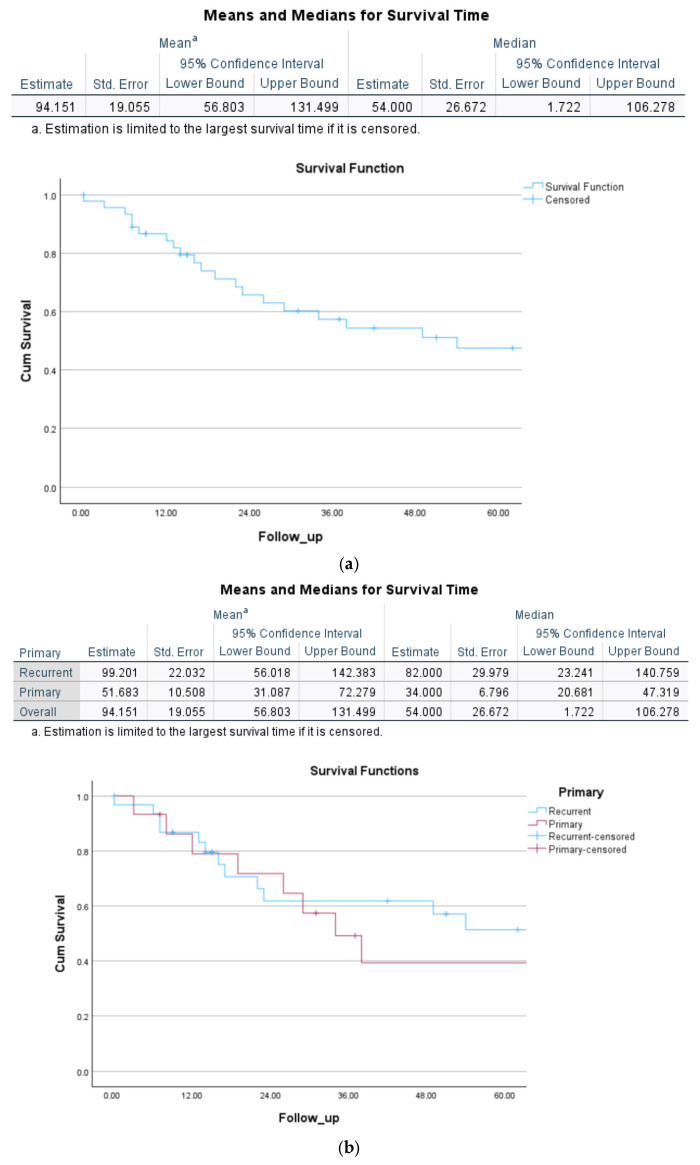
(**a**) Overall survival post curative intent (N = 46/47) pelvic exenterative surgery. Numbers at risk are shown below the *x*-axis at 0, 12, 24, 36, and 60 months. (**b**) Comparison of overall survival for primary vs. recurrent gynaecological tumours for curative intent PE (N = 46/47 cases, *p* > 0.05). Numbers at risk are shown below the *x*-axis at 0, 12, 24, 36, and 60 months.

The Kaplan–Meier curve for (curative intent N = 46/47) overall survival is demonstrated in Figure 2a and OS for recurrent vs. primary disease in Figure 2b.

## 4. Discussion

In this retrospective cohort study where we present our two-decade experience from a single tertiary centre, PE was associated with substantial perioperative morbidity but low mortality, alongside encouraging long-term oncological outcomes. Major complications occurred in approximately one-third of patients, while perioperative mortality remained low. Despite the heterogeneity of tumour types, a meaningful proportion of patients achieved disease control for at least 2 years, with over 40% remaining recurrence-free. Although there is no statistically significant difference in survival outcomes when comparing PE for primary vs. recurrent disease, there is a trend of longer survival in the recurrent disease group. Patterns of recurrence were predominantly distant, highlighting the disease biology. These findings support the role of PE as a vital treatment option in carefully selected patients.

### 4.1. Reported Morbidity and Mortality

PE remains associated with significant perioperative mortality despite advances in surgical and perioperative care. A recent meta-analysis by Esmailzadeh et al. reported mortality being as high as 0.21% intraoperatively; in-hospital mortality was 2.65%, and 30-day mortality approached 5.9%. Sepsis was identified as the leading cause of death [11]. Similarly, Di Donato et al. demonstrated that disease extent significantly influences perioperative outcomes, with pelvic sidewall involvement and TPE associated with increased 30-day mortality [6].

In our cohort, perioperative mortality was 2.1%, which lies within the lower range of the contemporary published reports. This likely reflects careful patient selection, delivery of care within a specialised multidisciplinary setting, and perioperative optimisation in a high-volume tertiary centre.

Our findings are also consistent with both UK and international series reporting substantial postoperative morbidity following PE. In our cohort, 31.9% of patients experienced major complications (Clavien–Dindo grade ≥ III), comparable to rates of approximately 30–40% reported by the PelvEx collaborative [12].

### 4.2. Oncological Outcomes Following Pelvic Exenteration

Despite this morbidity burden, oncological outcomes remain encouraging in selected patients. In the present study, the 5-year overall survival rate was 45.9%, which falls within the 30–60% range reported in the contemporary literature [6,13,14]. Similarly, a systematic review by Miri et al. reported a wide range of 5-year overall survival (6.0–64.6%), reflecting the role of heterogeneity in patient cohorts and disease characteristics [15].

The favourable survival outcomes observed in our cohort are likely attributable to a high rate of complete cytoreduction, with 83% of patients achieving clear (R0) resection margins. This is consistent with existing evidence identifying negative surgical margins as the most important determinant of long-term survival. Data from the PelvEx collaborative and COREPEX study confirm that margin status remains the strongest predictor of oncological outcomes following PE [8,12].

Additional prognostic factors influencing survival include nodal status and disease extent. The meta-analysis by Di Donato et al., including over 4000 patients, demonstrated that increasing rates of pelvic and para-aortic nodal involvement are associated with a significant negative impact on survival [6]. Pelvic sidewall involvement similarly confers a worse prognosis, reflecting the challenges of achieving clear margins in anatomically complex disease [16].

#### Recurrence Patterns

Recurrence following PE remains common and represents a major determinant of long-term outcomes. In our cohort, 41% of patients remained recurrence-free, while distant recurrence was the predominant pattern (51%), with local relapse occurring in only 9% of patients. When comparing the remission period between primary vs. recurrent tumour, there is a clear trend of longer mean remission period (42 vs. 1.6 months) in the recurrent cohort. This keeps with disease biology since a longer remission period (DFI prior to PE) is perceived as a good prognostic factor.

These findings are in keeping with contemporary evidence suggesting that, in modern series, distant metastases are the most frequent mode of failure following PE. In the COREPEX study, recurrence occurred in approximately 44% of patients, with distant relapse representing the most common pattern, followed by central pelvic recurrence [8]. Importantly, adverse prognostic factors included positive surgical margins, presence of lymphovascular space invasion, and para-aortic nodal disease, all of which were associated with poorer oncologic outcomes.

In contrast, UK-based data focusing on recurrent and previously treated disease have demonstrated a higher proportion of locoregional relapse. For example, Gomes et al. reported a recurrence rate of 60%, with most recurrences occurring within the pelvis. Notably, locoregional control was significantly improved in patients with clear surgical margins compared with those with involved or close margins, underscoring the critical importance of complete resection [16].

Taken together, these findings suggest that recurrence patterns are influenced by disease biology, prior treatment, and surgical factors. The relatively low rate of local recurrence observed in our cohort likely reflects effective surgical clearance, while the predominance of distant relapse highlights the ongoing challenge of systemic disease control.

The big disparity between distant recurrence (51%) and local relapse (9%) demands a fundamental rethink of post-PE surveillance and adjuvant strategy. Locoregional control is clearly achievable; systemic disease may not be. Conventional follow-up protocols centred on pelvic imaging are therefore insufficient for this population. Systemic cross-sectional imaging—CT chest–abdomen–pelvis or PET-CT—should be standard in the first 2–3 years post-surgery, when recurrence risk is greatest. Beyond surveillance, the predominance of distant failure raises an uncomfortable question: are we operating on patients with occult systemic disease? This warrants serious consideration of adjuvant systemic therapy following PE, particularly in those with high-risk features such as lymphovascular space invasion, nodal involvement, or close/involved margins. Prospective evaluation of adjuvant strategies in this setting is an ongoing subject of debate.

### 4.3. Follow-Up and Quality of Life

Optimal follow-up strategies following PE remain poorly standardised. In our cohort, the median follow-up was 26 months (mean follow-up 46 months with a SD of 51), with 41% of patients remaining recurrence-free during this period. The major difference between median and mean follow-up is attributed to the fact that case volume increased over the course of the last 5 years, hence the variation in follow-up duration. We also note that during the last 6-year period (2019–2024) the case volume tripled (N = 19) compared to the first 5-year interval (2004–2009, N = 6).

A systematic review by Siu et al. demonstrated considerable variability in follow-up protocols, with most centres adopting frequent clinical review every 3–4 months during the first 2–3 years, followed by less intensive surveillance thereafter. Imaging strategies varied widely and included CT, MRI, and PET imaging, often performed at regular intervals over 5–10 years [17].

Importantly, quality of life (QoL) outcomes following PE remain underreported. While some studies have utilised validated tools such as the EORTC QLQ-C30 and SF-36, there is a lack of standardisation and limited integration of functional and oncological outcomes which was supported by a recent systematic review [17]. Given the significant physical and psychological impact of this procedure, further research is required to better define long-term QoL and survivorship outcomes.

### 4.4. Future Directions: Role of Minimally Invasive and Robotic Approaches

Minimally invasive and robotic approaches to PE are emerging and may offer potential benefits in selected patients, including reduced blood loss, shorter hospital stays, and possibly lower perioperative morbidity [18,19]. While early reports are encouraging, there is a paucity of robust data on long-term oncological outcomes [20,21]. At our institution, the implementation of robotic PE is underway and represents an important step toward expanding surgical options, particularly for patients who may not be optimal candidates for open surgery.

### 4.5. Role of Radiotherapy Technique: Three-Dimensional Conformal Radiotherapy Versus Volumetric Arc Therapy

A proportion of patients in our cohort had received prior pelvic radiotherapy, reflecting the natural history of recurrent gynaecological malignancies. Radiotherapy technique—particularly Three-Dimensional Conformal Radiotherapy (3DCRT) versus Volumetric Modulated Arc Therapy (VMAT)—may influence both oncological and surgical outcomes [22,23,24]. VMAT, a form of intensity-modulated radiotherapy (IMRT), enables highly conformal dose delivery, facilitating target coverage while reducing exposure to adjacent organs at risk, including the bowel, bladder, and rectum. IMRT-based techniques have been associated with lower rates of acute and late gastrointestinal and genitourinary toxicity compared with 3DCRT in gynaecological cancers [22].

This is relevant to PE, where prior irradiation can compromise tissue planes, increasing operative complexity and the risk of complications such as fistulae, bowel injury, and impaired wound healing. More conformal techniques may better preserve tissue integrity and potentially reduce postoperative morbidity, although this remains unproven. To our knowledge, there is no prospective trial to directly compare PE outcomes according to radiotherapy technique, representing an important area for future research. Incorporation of detailed radiotherapy parameters—including technique, dose, and treatment fields—into prospective PE registries may enable more robust evaluation of these associations.

### 4.6. Strengths

The strengths of this study include its long study period, reflecting two decades of experience within our tertiary centre, and the inclusion of a well-defined cohort of patients undergoing PE. Detailed perioperative and oncological data allowed for comprehensive evaluation of morbidity, recurrence patterns, and survival outcomes.

### 4.7. Limitations

This study is limited by its retrospective design and therefore no formal power calculation was performed. The small sample size precluded any regression modelling and therefore analysis was limited to descriptive statistics. The heterogeneity of tumour types and indications for surgery may also limit the ability to draw conclusions for specific disease patterns. Additionally, data on quality of life and functional outcomes were not available, representing an important gap given the impact of PE on patients. The relatively small sample size further limits the ability to perform subgroup analyses. We do acknowledge the possibility of PE cases having been missed during our identification process, especially during the first decade of the covered period. We also note that almost half of our cohort was identified over the course of the last 6 years. This is mainly because our centre tripled its PE surgical volume over the course of the last 6 years. Hence, important follow-up outcomes are yet to be identified and analysed.

### 4.8. Future Endeavours

Pelvic exenteration (PE) is a complex surgical procedure that requires a high level of expertise and should be performed in centres with appropriate experience. Our findings support the notion that, within our institution, PE can be undertaken safely in a carefully selected cohort of women. Prospective registration of patients in a predesigned database represents an important step toward improving data quality and consistency. Furthermore, national and international collaboration between specialised PE centres will facilitate the exchange of knowledge and experience. Such collaborative efforts may also enable the development of shared data registries, ultimately allowing for more robust analyses and the generation of clinically meaningful conclusions.

## 5. Conclusions

PE remains a complex procedure associated with significant but acceptable morbidity and mortality in experienced centres. In carefully selected patients, it offers meaningful oncological benefit and durable disease control. Outcomes are strongly influenced by patient selection, surgical expertise, and multidisciplinary care.

## Figures and Tables

**Table 1:** Baseline cohort characteristics and outcomes.

Variable	Value
Baseline characteristics	
Age at diagnosis (years) (median, IQR)	55 (49–66)
Age at surgery (years) (median, IQR)	60 (50–68)
BMI (kg/m^2^) (median, IQR)	28.1 (19.7–53.0)
Performance status, n (%)	
PS 0	37 (78.7%)
PS 1	10 (21.3%)
Co-morbidities, n (%)	
Vasculopathy	4 (8.51%)
Hypertension	20 (42.6%)
Diabetes mellitus	6 (12.8%)
Pathology, n (%)	
Vulval	11 (23.4%)
Endometrial	17 (36.2%)
Ovarian	7 (14.9%)
Cervical	5 (10.6%)
Vaginal	7 (14.9%)
Disease status, n (%)	
Primary disease	15 (32.0%)
Recurrent disease	32 (68.0%)
Previous treatment n (%)	
TAH ± PLDN	6 (12.8%)
TAH BSO, brachytherapy ± external beam radiotherapy	7 (14.9%)
Radical hysterectomy with adjuvant therapy	3 (6.4%)
Radiotherapy and/or chemotherapy	8 (17%)
Intent of surgery, n (%)	
Palliative	1 (2%)
Curative	46 (98%)
Intraoperative details/outcomes n (%)	
TPE	18 (38%)
APE	15 (32%)
PPE	14 (30%)
Flap reconstruction	10 (21%)
Urinary diversion	32 (68%)
EBL L (median, IQR)	1.5 (IQR 0.85–2.0)
Operative time, minutes	391 (IQR 313–482)
Postoperative outcomes	
HDU stay (days median, IQR)	4 (IQR 2–5)
Length of stay (days median, IQR)	17 (IQR 13–31)
CD I–II n (%)	25 (53.2%)
CD III–V n (%)	15 (31.9%)
Oncological outcomes	
Disease-free interval for recurrent tumours prior to PE (years) (median, IQR)	2 (0–2)
Median follow-up (months, IQR)	26 (12–64)
Local recurrence n (%)	4 (9%)
Distant recurrence n (%)	24 (51%)
Curative intent (N = 46/47) PE survival (mean, CI)	94 (57–131)
Primary tumour (N = 15) survival (mean, CI)	51.68 (31–72)
Recurrent tumours (N = 32) (mean, CI)	99.2 (56–142)

BMI: body mass index, PS: performance status, TAH: total abdominal hysterectomy, CI: confidence interval, PLDN: pelvic lymph node dissection, BSO: bilateral salpingoophorectomy, TPE: total pelvic exenteration, APE: anterior pelvic exenteration, PPE: posterior pelvic exenteration, EBL: estimated blood loss, HDU: high dependency unit, CD: Clavien–Dindo, IQR: interquartile range.

## Data Availability

Data available on request due to restrictions (e.g., privacy, legal or ethical reasons).

## Abbreviations

The following abbreviations are used in this manuscript:

BMI	Body Mass Index
PS	Performance Status
TAH	Total Abdominal Hysterectomy
PLDN	Pelvic Lymph Node Dissection
BSO	Bilateral Salpingoophorectomy
TPE	Total Pelvic Exenteration
APE	Anterior Pelvic Exenteration
PPE	Posterior Pelvic Exenteration
EBL	Estimated Blood Loss
HDU	High Dependency Unit
CD	Clavien–Dindo
IQR	Interquartile Range
